# The association between physical exercise and health care seeking behavior in older adults: the mediating role of self-perceived health

**DOI:** 10.3389/fpubh.2025.1566321

**Published:** 2025-05-09

**Authors:** Jian Li, Xing Zhao, Hongjuan Li

**Affiliations:** ^1^Faculty of Economics and Management, Shanghai University of Sport, Shanghai, China; ^2^Faculty of Mathematical and Information Science, Shaoxing University, Shaoxing, China

**Keywords:** physical exercise, health care seeking behavior, older adults, physical health, mental health, mediating analysis, heterogeneity analysis

## Abstract

**Background:**

As global aging accelerates, the health of older adults has become a critical issue. Physical exercise has long been recognized for positive effects on health, particularly among the older adults. However, the specific impact of physical activity on health care seeking behavior, as well as the mechanisms underlying this relationship, remains insufficiently explored. This study investigates the effect of physical activity on health care seeking behavior in older adults and explores the mediating roles of physical and mental health.

**Methods:**

Using data from the 2021 China General Social Survey (CGSS), this study employs regression analysis, and mediation analysis to examine how physical activity influences the health care seeking behavior of older adults. The analysis includes the evaluation of physical and mental health as mediators in this relationship. Additionally, heterogeneity analysis is conducted to explore differences across various age groups, and robustness tests are performed using lasso regression and ridge regression to ensure the stability of the results.

**Results:**

This study finds that physical activity negatively influences health care seeking behavior in older adults (*β* = −0.100, *p* < 0.001, 95%CI = [−0.154, −0.046]), with physical health (β = −0.026, 95%CI = [−0.087, −0.010]) and mental health (β = −0.008, 95%CI = [−0.074, −0.001]) acting as significant mediators. The effect of physical activity on health care seeking behavior is particularly pronounced in older adults aged 60–69 (*β* = −0.083, *p* = 0.027, 95%CI = [−0.158, −0.009]) and 70–79 (β = −0.154, *p* = 0.001, 95%CI = [−0.248, −0.060]). Robustness tests confirm the stability and reliability of the findings, with lasso regression and ridge regression further supporting the conclusions.

**Conclusion:**

The findings highlight the importance of physical activity in reducing health care seeking behavior and health management in older adults. By improving both physical and mental health, physical exercise can effectively reduce the medical seeking behavior of older adults. This study provides valuable insights for developing targeted health interventions and policies aimed at improving the health of older adults.

## 1 Introduction

With the accelerated global aging process, the health issues of the older adults have become a focal point of social attention. They face various health challenges, including chronic diseases, cognitive decline, and mental health issues. Recent projections suggest that by 2050, the number of older adults will exceed 2 billion globally, leading to increased pressure on healthcare systems (1). Therefore, improving the health and quality of life of older adults has become a significant public health concern. In recent years, an increasing number of studies have recognized the importance of physical activity and exercise for health, especially for older adults. Research has shown that regular physical exercise not only helps older adults maintain physical health and reduce the occurrence of chronic diseases but also significantly improves mental health and quality of life (2–4). Studies have demonstrated that moderate-intensity physical activity can reduce the risks of cardiovascular diseases, diabetes, and some cancers, and can also delay the onset of dementia and other cognitive impairments (5). Therefore, how to promote the health of older adults through effective physical exercise, particularly in terms of enhancing health care seeking behavior, has become a topic worth exploring.

Health care seeking behavior refers to the various medical actions and behaviors individuals take when facing health issues, including seeking medical care, receiving treatment, and utilizing medical resources. Previous studies have emphasized that health care seeking behavior is influenced by a complex interplay of biological, psychological, and social factors (6). Studies have found that health care seeking behavior is closely related to an individual’s health status, mental state, and lifestyle (7–9). For older adults, physical activity not only has a direct impact on physical health but may also indirectly promote changes in health care seeking behavior by improving mental health and enhancing self-efficacy (10). However, the mechanisms through which physical activity affects health care seeking behavior in older adults remain unclear, particularly in terms of the mediating roles of physical and mental health. To address this issue, the present study aims to investigate the impact of physical activity on the health care seeking behavior of older adults and further analyze the mediating roles of physical and mental health. Based on data from the 2021 China General Social Survey (CGSS), this study employs statistical methods such as regression analysis, and mediation analysis to examine the impact of physical activity on the health care seeking behavior of the older adults and the underlying mechanisms. Specifically, the study first examines the relationship between physical activity and health care seeking behavior in older adults, proposing that physical activity may influence health care seeking behavior by improving physical and mental health. Second, the study focuses on the mediating roles of physical health and mental health in the relationship between physical activity and health care seeking behavior, further validating the beneficial role of exercise in promoting health management among older adults. The mental health benefits of physical activity, such as reduced depression and anxiety, may lead to more proactive health-seeking behaviors (11). Lastly, this study conducts heterogeneity analysis to explore the differences in the impact of physical activity on health care seeking behavior across different age groups of older adults, providing evidence for targeted health intervention measures.

The innovative aspect of this study lies in its comprehensive investigation of the impact of physical exercise on health care seeking behavior from the dual perspectives of physical and mental health. By examining how physical exercise influences health care seeking behavior through improvements in both physical and mental health, this study provides a new theoretical perspective and support for future interventions and policies aimed at the health of older adults. Furthermore, the findings of this study are of great practical significance for promoting targeted and personalized health interventions for older adults. Overall, through a comprehensive exploration of the impact of different frequencies and intensities of physical activity on health care seeking behavior among older adults of all ages, this study provides important theoretical and empirical support for promoting the health of older adults and improving health care seeking behavior.

## 2 Materials and methods

### 2.1 Data processing

**Figure 1:**
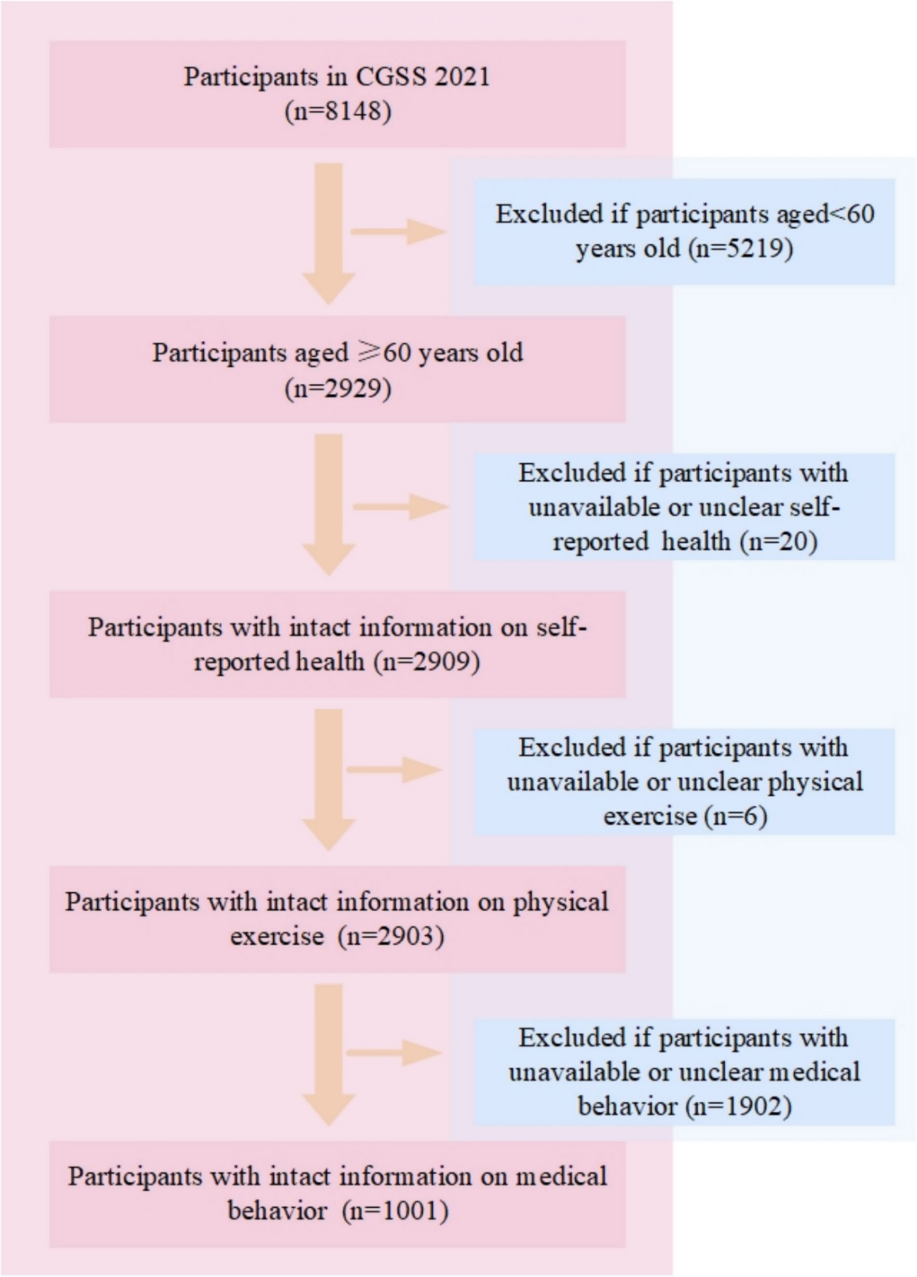
Flowchart of the inclusion of the study participants.

The data is taken from the 2021 Chinese General Social Survey (CGSS), which is the earliest national, comprehensive, and continuous academic survey project in China. The CGSS adopts a multi-stage stratified probability sampling design and covers over 10,000 households across all provinces, municipalities, and autonomous regions in China, systematically and comprehensively collecting data at the social, community, household, and individual levels. The CGSS 2021 consists of three parts: the core module, the household questionnaire module, and the social network module, containing more than 800 variables. The data quality is strictly controlled and has good representativeness and reliability. As the 2021 CGSS includes rich data on factors influencing residents’ health, data from this year was selected for this study. The data was officially released on March 31, 2023. The original dataset contained 8,148 participants. In this study, the research team screened the original samples based on the research objectives, and the sample screening, data cleaning, and statistical analysis were completed by researchers with a background in social science quantitative research, ensuring the scientific nature of the data processing process and the credibility of the results. The data screening process followed a series of inclusion and exclusion criteria to ensure the focus of the study was on older adults and related health information. As shown in Figure 1. First, participants under the age of 60 were excluded, leaving 2,929 participants aged 60 and above. Next, participants with incomplete or unclear self-reported health information (*n* = 20) were excluded, leaving 2,909 participants with valid self-reported health data. Subsequently, participants with unavailable or unclear physical exercise information (*n* = 6) were excluded, leaving 2,903 participants with valid physical exercise data. Finally, participants without valid or clear health care seeking behavior data (*n* = 1,902) were excluded, resulting in a final sample of 1,001 participants for analysis. The final dataset of 1,001 older adults was used to explore the relationship between physical exercise, self-perceived health, and health care seeking behavior. The data was processed and cleaned to address missing values and ensure consistency of the variables used for analysis.

### 2.2 Variables description

#### 2.2.1 Independent variable

“Physical exercise” was measured using the question: “In the past year, did you regularly engage in physical exercise during your leisure time?” Respondents were asked to choose from five options, each corresponding to a numerical value: a response of “never” was assigned a value of 1, indicating no participation in physical exercise; “occasionally” was assigned a value of 2, reflecting infrequent engagement; “sometimes” received a value of 3, indicating moderate frequency; “often” was assigned a value of 4, representing frequent participation; and “always” was given a value of 5, indicating regular and consistent physical exercise. The higher the numerical value, the more frequent the engagement in physical exercise, signifying a higher level of physical activity during leisure time.

In the robustness test, “high-intensity exercise” and “sports venue” were selected as independent variables. “high-intensity exercise” was measured by the question: “Do you regularly engage in at least 20 min of physical exercise that can make you sweat or breathe faster? “The responses were assigned a value of 1–5 from “never” to “every day.” The variable of “sports venue” was asked by the question “Is your place of residence suitable for physical exercise (such as jogging, walking)?” Answer “no” was assigned a value of 1, answer “yes” was assigned a value of 2.

#### 2.2.2 Dependent variable

“Health care seeking behavior” was measured using the question: “In the past year, how frequently did you seek medical care? (This refers to seeking medical treatment due to your own illness or injury, either alone or accompanied by family members, excluding visits to accompany or visit other patients.)” Respondents were asked to choose from five options, each corresponding to a numerical value: a response of “always” was assigned a value of 5, indicating frequent medical visits; “often” was assigned a value of 4, reflecting a high frequency of visits; “sometimes” received a value of 3, indicating occasional visits; “occasionally” was assigned a value of 2, representing infrequent medical care-seeking behavior; and “never” was given a value of 1, indicating no visits to medical facilities in the past year. The higher the numerical value, the more frequent the engagement in health care seeking behavior, indicating a higher level of medical care-seeking behavior.

#### 2.2.3 Mediator variables

“Self-perceived physical health” was measured using the question: “How would you rate your current physical health?” Respondents were asked to choose from five options, each corresponding to a numerical value: a response of “very poor” was assigned a value of 1, indicating a very poor self-assessment of physical health; “poor” was assigned a value of 2, reflecting a poor perception of physical health; “fair” received a value of 3, indicating an average or neutral assessment of physical health; “good” was assigned a value of 4, representing a good self-assessment; and “very good” was given a value of 5, indicating a very positive perception of physical health. The higher the numerical value, the better the individual’s self-perceived physical health.

Similarly, the mediator variable “Self-perceived mental health” was measured using the question: “How would you rate your current mental health?” The responses were assigned values in the same way: “very poor” was given a value of 1, indicating a very poor self-assessment of mental health; “poor” was assigned a value of 2, reflecting a poor perception of mental health; “fair” received a value of 3, indicating an average or neutral assessment; “good” was given a value of 4, representing a good self-assessment; and “very good” was assigned a value of 5, indicating a very positive perception of mental health. The higher the numerical value, the better the individual’s self-perceived mental health.

#### 2.2.4 Control variables

In this study, to reduce confounding effects and better isolate the impact of physical activity, the control variables were categorized and assigned values as follows: Age was divided into three groups: 1 = 60–69 years old, 2 = 70–79 years old, and 3 = 80 years old and above. Gender was coded as 1 = male and 2 = female. Residence was classified as 1 = rural and 2 = urban. Education level was categorized into 1 = illiterate, 2 = primary education, 3 = middle school, and 4 = high school and above. Marital status was represented as 1 = with partner and 2 = without partner. Income was treated as a continuous variable, using the natural logarithm of the respondent’s total annual income. Chronic disease was coded as 0 = no chronic disease, 1 = at least one chronic disease (a total of 14 chronic non communicable diseases).

### 2.3 Statistical analysis

Data analysis was conducted using R 4.4.1. Descriptive statistics were first performed to summarize the sample characteristics, including means and standard deviations for continuous variables and frequencies and percentages for categorical variables. To examine the relationship between physical exercise and health care seeking behavior, a series of regression analyses were performed (12). Regression models were adjusted for potential confounders, including demographic variables such as age, gender, and residence, as well as health status variables, to ensure that the observed relationships were not influenced by these factors. In addition to the basic regression analysis, mediation analysis was conducted to investigate the indirect effects of physical health and psychological health on the relationship between physical exercise and health care seeking behavior (13). This was done using a bootstrapping method to assess the significance of the indirect effects. To test the robustness of the findings, lasso and ridge regression were employed as part of the sensitivity analysis (14). Finally, to explore age-related differences in the impact of physical exercise on health care seeking behavior, heterogeneity analysis was conducted by stratifying the sample into different age groups.

## 3 Research results

### 3.1 Descriptive statistics and correlation analysis of each variable

The baseline characteristics of the participants are summarized in Table 1. The study included a total of 1,001 participants. The majority of the participants were aged between 60 and 69 years old (51.55%). The gender distribution was nearly equal, with 49.35% being male and 50.65% female. Most participants resided in rural areas (58.64%). In terms of education, 22.08% were illiterate, 33.57% had primary education, 24.68% had middle school education, and 19.68% had high school or higher education. The majority of participants were married or had a partner (72.43%). The average natural logarithm of income was 7.34. Most older adults (61.14%) suffer from chronic diseases. Regarding physical exercise habits, 50.75% of the participants reported exercising often, while 3.40% never exercised. The sports venue environment was considered adequate by 73.83% of the participants. 8.89% of older adult people engage in high-intensity exercise every day that can make them sweat or breathe faster for more than 20 min. Health care seeking behavior was reported as always by 3.80% of the participants, often by 12.19%, sometimes by 35.16%, occasionally by 20.18%, and never by 28.67%. Health check-ups were not conducted by 57.24% of the participants, while 42.76% had undergone health check-ups. Self-perceived physical health was rated as very poor by 8.79%, poor by 21.98%, fair by 31.97%, good by 27.37%, and very good by 9.89%. Self-perceived mental health was rated as very poor by 3.80%, poor by 10.59%, fair by 23.58%, good by 25.17%, and very good by 36.86%.

**Table 1:** Baseline characteristics of participants.

Characteristic	Groups	*n* (Mean)	% (S.E.)
		1,001	100.00
Age	1 = 60–69 years old	516	51.55
	2 = 70–79 years old	365	36.46
	3 = 80 years old and above	120	11.99
Gender	1 = male	494	49.35
	2 = female	507	50.65
Residence	1 = rural	587	58.64
	2 = urban	414	41.36
Education	1 = illiterate	221	22.08
	2 = primary	336	33.57
	3 = middle	247	24.68
	4 = high and above	197	19.68
Marital status	1 = with partner	725	72.43
	2 = without partner	276	27.57
Ln (income)	Continuous variable	7.34	4.54
Chronic disease	0 = no	389	38.86
	1 = yes	612	61.14
Physical exercise	1 = never	34	3.40
	2 = occasionally	141	14.09
	3 = sometimes	87	8.69
	4 = often	508	50.75
	5 = always	231	23.08
Sports venue environment	1 = no	262	26.17
	2 = yes	739	73.83
High-intensity exercise	1 = never	281	28.07
	2 = once a month	225	22.48
	3 = several times a month	234	23.38
	4 = several times a week	172	17.18
	5 = every day	89	8.89
Health care seeking behavior	1 = never	287	28.67
	2 = occasionally	202	20.18
	3 = sometimes	352	35.16
	4 = often	122	12.19
	5 = always	38	3.80
Self-perceived physical health	1 = very poor	88	8.79
	2 = poor	220	21.98
	3 = fair	320	31.97
	4 = good	274	27.37
	5 = very good	99	9.89
Self-perceived mental health	1 = very poor	38	3.80
	2 = poor	106	10.59
	3 = fair	236	23.58
	4 = good	252	25.17
	5 = very good	369	36.86

**Figure 2:**
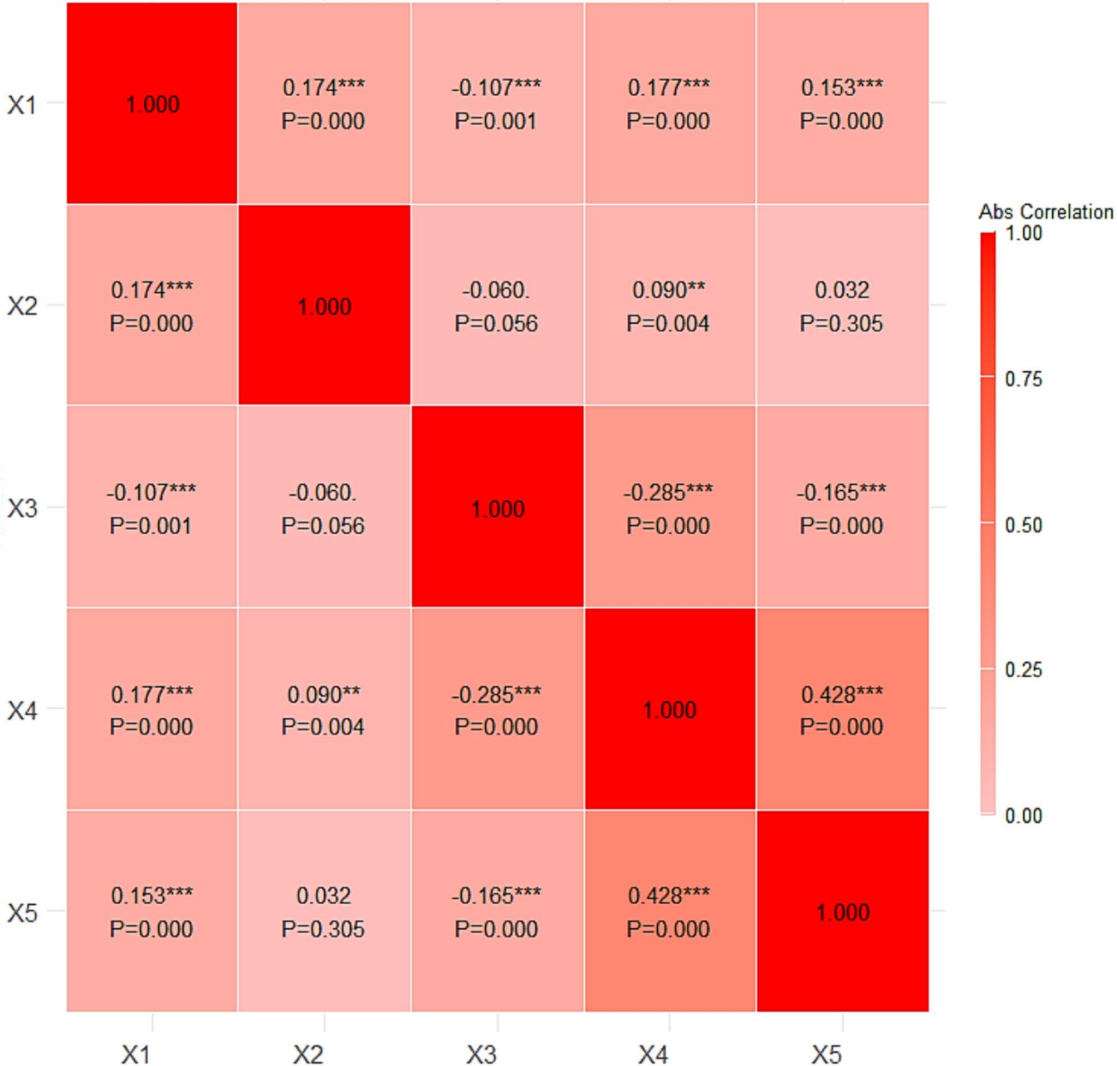
Intercorrelation matrix. X1, physical exercise; X2, sports venue; X3, health care seeking behavior; X4, self-perceived physical health; X5, self-perceived mental health. ^**^p < 0.01, ^***^p < 0.001.

The intercorrelation matrix displayed in Figure 2 presents the significant correlations among the variables. Physical exercise (X1) is positively associated with sports venue (X2), as indicated by a correlation coefficient of 0.174, which is significant at the <0.001 level. Physical exercise (X1) is negatively associated with health care seeking behavior (X3), as indicated by a correlation coefficient of −0.107, which is significant at the 0.001 level. Furthermore, physical exercise (X1) shows a positive association with self-perceived physical health (X4), with a correlation coefficient of 0.177, significant at <0.001 level, and with self-perceived mental health (X5), with a correlation coefficient of 0.153, significant at the 0.000 level. Health care seeking behavior (X3) is also negatively associated with self-perceived physical health (X4), with a correlation coefficient of −0.285, significant at the 0.000 level, and with self-perceived mental health (X5), with a correlation coefficient of −0.165, significant at the 0.000 level. Additionally, self-perceived physical health (X4) is strongly associated with self-perceived mental health (X5), with a high correlation coefficient of 0.428, significant at the 0.000 level.

### 3.2 Impact of physical exercise on health care seeking behavior

The regression results presented in Table 2 examine the impact of physical exercise on the health care seeking behavior of older adults across four models. In Model 1, which includes only physical exercise as the independent variable, the effect is significantly negative (*β* = −0.092, *p* < 0.01), with a 95% confidence interval of [−0.145, −0.039]. This indicates that older adults who engage in more frequent physical exercise tend to exhibit lower levels of health care seeking behavior, suggesting that physical activity may reduce the need for medical services. Model 2 incorporates chronic diseases along with demographic controls. The negative association between physical exercise and health care seeking behavior remains significant (*β* = −0.087, *p* < 0.001), with a confidence interval of [−0.151, −0.053]. Chronic diseases show a strong positive association with health care seeking behavior (β = 0.762, *p* < 0.001), indicating that older adults with chronic conditions are more frequent users of medical services. In addition, age (*β* = 0.093, *p* < 0.01), gender (β = 0.209, *p* < 0.05), and residence (β = 0.306, *p* < 0.01) are significantly positively associated with health care seeking behavior, suggesting that older adults, males, and urban residents are more likely to seek medical care. In Model 3, self-perceived physical health is added. The effect of physical exercise remains significantly negative (*β* = −0.084, *p* < 0.01), with a confidence interval of [−0.138, −0.030]. Self-perceived physical health is significantly negatively associated with health care seeking behavior (*β* = −0.278, *p* < 0.001), indicating that older adults who perceive their physical health more positively exhibit lower levels of health care seeking behavior. Model 4 introduces self-perceived mental health. The effect of physical exercise remains robust and significantly negative (β = −0.100, *p* < 0.001), with a confidence interval of [−0.154, −0.046]. Self-perceived mental health is also negatively associated with health care seeking behavior (β = −0.136, *p* < 0.001), suggesting that individuals with better mental health perceptions are less likely to seek medical care. The adjusted R-squared values increase from 0.036 in Model 1 to 0.185 in Model 4, indicating that the models explain a larger proportion of the variance in health care seeking behavior as more variables are included.

**Table 2:** Regression results of the impact of physical exercise on the health care seeking behavior of older adults.

Factors	Model 1	Model 2	Model 3	Model 4
Physical exercise	−0.092^**^ [−0.145, −0.039]	−0.087^***^ [−0.151, −0.053]	−0.084^**^ [−0.138, −0.030]	−0.100^***^ [−0.154, −0.046]
Age		0.093^**^ [0.046, 0.231]	0.083^*^ [0.053, 0.219]	0.095^*^ [0.043, 0.233]
Gender		0.209^*^ [0.016, 0.401]	0.189^*^ [0.001, 0.379]	0.184^*^ [0.001, 0.361]
Residence		0.306^**^ [0.085, 0.526]	0.333^**^ [0.117, 0.550]	0.331^**^ [0.111, 0.550]
Education		0.008 [−0.098, 0.114]	0.014 [−0.090, 0.118]	0.018 [−0.088, 0.123]
Marital status		0.064 [−0.148, 0.276]	0.061 [−0.148, 0.269]	0.045 [−0.167, 0.256]
Ln (income)		0.014 [−0.009, 0.036]	0.015 [−0.007, 0.037]	0.016 [−0.007, 0.038]
Chronic diseases		0.762^***^ [0.574, 0.949]	0.489^***^ [0.285, 0.693]	0.683^***^ [0.491, 0.876]
Self-perceived physical health			−0.278^***^ [−0.369, −0.188]	
Self-perceived mental health				−0.136^***^ [−0.219, −0.053]
Constant	3.386^***^ [3.214, 3.557]	1.862^***^ [1.323, 2.401]	2.809^***^ [2.197, 3.422]	2.393^***^ [1.767, 3.020]
Adj R-squared	0.036	0.177	0.212	0.185

^*^p < 0.05, ^**^p < 0.01, ^***^p < 0.001.

### 3.3 Mediation effects of physical and mental health on health care seeking behavior

**Figure 3:**
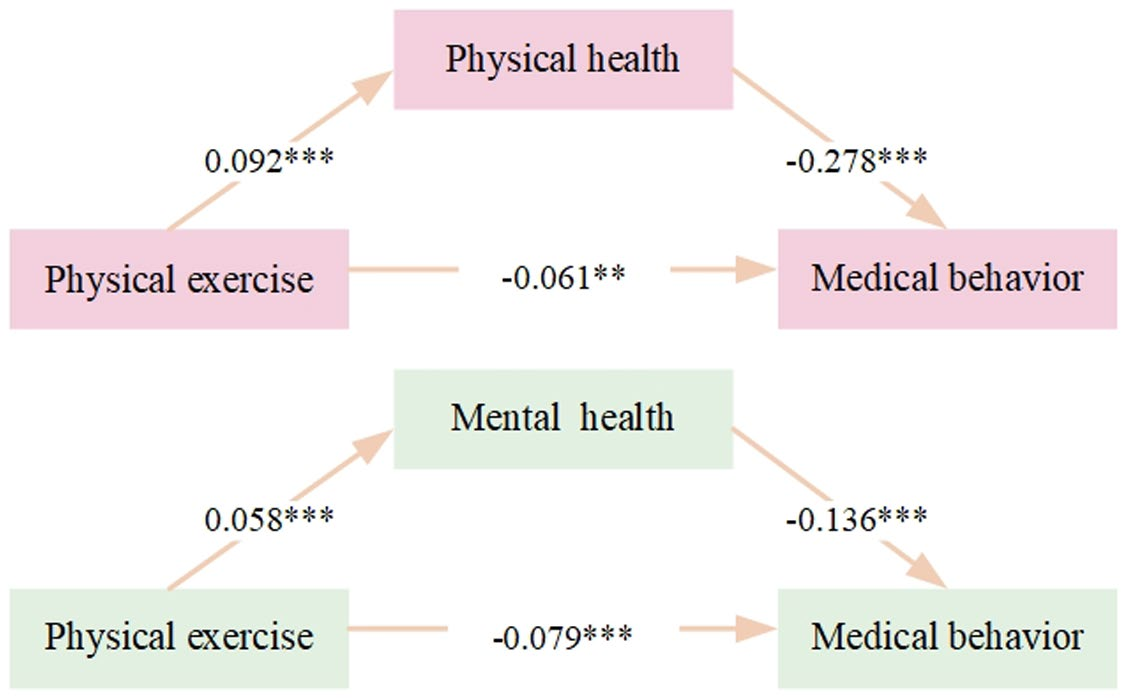
Mediation effect model diagram. Age, gender, residence, marital status, individual income, and chronic diseases have been controlled. ^**^p < 0.01, ^***^p < 0.001.

The mediation effect model depicted in Figure 3 illustrates the relationship between physical exercise and health care seeking behavior, with physical health and mental health serving as potential mediators. The model is controlled for age, gender, residence, marital status, individual income, and chronic diseases. In Path 1, physical exercise has a significant positive effect on physical health (*β* = 0.092, *p* < 0.001), which in turn significantly influences health care seeking behavior (*β* = −0.278, *p* < 0.001). The direct effect of physical exercise on health care seeking behavior is also significant (*β* = −0.061, *p* < 0.01), indicating that physical exercise influences health care seeking behavior both directly and indirectly through physical health. In Path 2, physical exercise has a significant positive effect on mental health (β = 0.058, *p* < 0.001), and mental health significantly influences health care seeking behavior (β = −0.136, *p* < 0.001). The direct effect of physical exercise on health care seeking behavior remains significant (β = −0.079, *p* < 0.01), suggesting that mental health also mediates the relationship between physical exercise and health care seeking behavior, although the direct effect is more pronounced. The bootstrap test results in Table 3 show that the direct effect of physical exercise on health care seeking behavior, mediated by physical health, is −0.061 (BootSE = 0.027, 95% CI = [−0.103, −0.025]), accounting for 70.11% of the total effect. The indirect effect, mediated by physical health, is −0.026 (BootSE = 0.013, 95% CI = [−0.087, −0.010]), representing 29.89% of the total effect. For the pathway mediated by mental health, the direct effect of physical exercise on health care seeking behavior is −0.079 (BootSE = 0.027, 95% CI = [−0.126, −0.019]), accounting for 90.80% of the total effect. The indirect effect, mediated by mental health, is −0.008 (BootSE = 0.002, 95% CI = [−0.074, −0.001]), representing 9.20% of the total effect.

**Table 3:** Bootstrap mediation effect test of physical health and mental health.

Effect path	Coef.	BootSE	LLCI	ULCI	Effect ratio
Direct effect (physical exercise → health care seeking behavior)	−0.061	0.027	−0.103	−0.025	70.11%
Indirect effect (physical exercise → physical health → health care seeking behavior)	−0.026	0.013	−0.087	−0.010	29.89%
Direct effect (physical exercise → health care seeking behavior)	−0.079	0.027	−0.126	−0.019	90.80%
Indirect effect (physical exercise → mental health → health care seeking behavior)	−0.008	0.002	−0.074	−0.001	9.20%

The bias-corrected percentile bootstrap method was used, with 5,000 repeated samples, to test the mediating effects, and the mediating effect model was based on Model 4 of the Process component.

### 3.4 Robustness and heterogeneity test results

**Figure 4:**
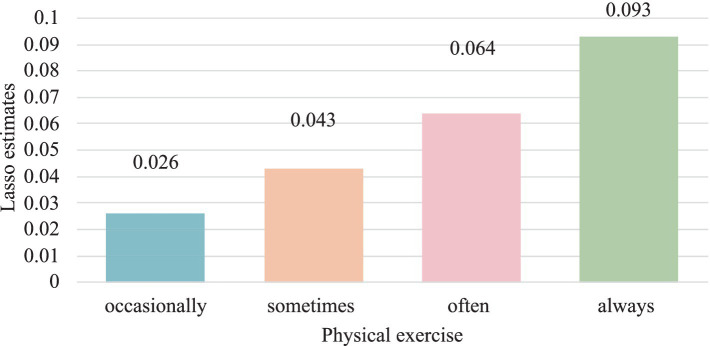
Impact of physical exercise on health care seeking behavior (Abs value).

The robustness test results in Table 4, which replace the variable “physical exercise” with “sports venue,” show a significant negative effect (*β* = −0.157, *p* < 0.05) on health care seeking behavior, consistent with the baseline regression results from Table 2. Furthermore, when replacing the variable of “physical exercise” with the variable of “high-intensity exercise,” it was found that older adults with higher exercise intensity visited medical institutions less frequently, which is also consistent with the results of basic research. This substitution method confirms the stability of the impact of physical exercise on health care seeking behavior, indicating good robustness. Furthermore, adopting a dual machine learning model (DML) to examine the impact of physical exercise on health care seeking behavior. The dual machine learning model combines traditional econometric methods and machine learning techniques, relaxes the assumptions about the intrinsic correlation of data, allows for more flexible functional forms, and has better stability. According to Table 5 and Figure 4, physical exercise has a gradually increasing negative effect on health care seeking behavior of older adults. Compared to never engaging in physical exercise, older adults who exercise more frequently tend to seek medical attention less frequently. Comparing the results of the dual machine learning model and the basic regression model, there is not much difference between the two. Overall, the research conclusions of the benchmark regression model are stable.

**Table 4:** Regression results of the impact of sports venue on the health care seeking behavior of older adults.

Factors	Coef.	S.E.	*t*	*p*	95%CI	
Sports venue	−0.157	0.023	−1.48	0.038^*^	−0.364	−0.103
_cons	3.554	0.363	9.79	<0.001^***^	2.842	4.267
High-intensity exercise	−0.045	0.016	−1.06	0.007^**^	−0.215	−0.014
Constant	3.384	0.321	10.52	<0.001^***^	2.753	4.015

Age, gender, residence, marital status, individual income, and chronic diseases have been controlled. ^*^p < 0.05, ^**^p < 0.01, ^***^p < 0.001.

**Table 5:** The dual machine learning model results of physical exercise and health care seeking behavior.

Factors	Lassocv	Ridgecv
Physical exercise (occasionally)	−0.026^***^	−0.034^***^
Sometimes	−0.043^***^	−0.048^***^
Often	−0.064^***^	−0.067^***^
Always	−0.093^***^	−0.095^***^
Age	0.086^**^	0.093^**^
Gender	0.194^*^	0.192^*^
Residence	0.300^**^	0.271^**^
Education	0.002	0.006
Marital status	0.054	0.065
Ln (income)	0.012	0.013
Chronic diseases	0.751^***^	0.691^***^

^*^p < 0.05, ^**^p < 0.01, ^***^p < 0.001.

Table 6 presents the heterogeneity analysis of the relationship between physical exercise and health care seeking behavior among different age groups of older adults. The results indicate significant differences in the impact of physical exercise on health care seeking behavior across age groups. The Chi-square test for heterogeneity is significant [χ^2^(*p*) = 13.35, *p* < 0.001]. For the age group of 60–69 years old, physical exercise has a negative effect on health care seeking behavior (*β* = −0.083, *p* < 0.05). The effect is stronger for the 70–79 years old age group (β = −0.154, *p* < 0.001), suggesting that physical exercise may be more beneficial for this age group in reducing health care seeking behavior. The effect for those aged 80 and above is not statistically significant (β = −0.112, *p* = 0.076).

**Table 6:** Heterogeneity analysis of physical exercise and health care seeking behavior among older adults of different age groups.

Groups	Coef.	Robust S.E.	*Z*	*p*	95%CI	
60–69 years old	−0.083	0.038	−2.21	0.027^*^	−0.158	−0.009
70–79 years old	−0.154	0.048	−3.21	0.001^**^	−0.248	−0.060
80 years old and above	−0.112	0.076	−1.49	0.137	−0.261	0.036
Chi2(*P*)	13.35(<0.001)					

^*^p < 0.05, ^**^p < 0.01.

## 4 Discussion

### 4.1 The impact of physical exercise on health care seeking behavior

The inverse relationship between physical exercise and health care seeking behavior observed in our study is a significant finding. Our results suggest that older adults who engage in regular physical exercise may have a reduced need for medical services, and the higher the frequency of exercise, the weaker their dependence on medical services (β = −0.100, *p* < 0.001). It is possibly due to a decrease in the incidence of preventable diseases and an increase in self-efficacy in managing their health. This aligns with the findings of a longitudinal study by Amanda et al. (15), which reported that older adults with higher physical activity levels had lower rates of hospital admissions. Although the regression results demonstrate that physical activity exerts a statistically significant effect on health care seeking behavior among older adults, the relatively narrow confidence interval indicates that the effect size is modest. This attenuated magnitude may be attributable to the inherent complexity of individual behavioral patterns and the multifactorial nature of healthcare-seeking behaviors, which are often influenced by variables such as socioeconomic status, accessibility of medical resources, and the burden of chronic conditions. In addition, the study by Liu et al. (16) demonstrated that high-intensity physical exercise significantly reduces healthcare expenditures among older adults, with a robust negative association (*β* = −0.0192, *p* < 0.01), which aligns with this study. Moreover, the current results also resonate with the concept of autogenic health, as proposed by Antonovsky (17), which emphasizes the importance of general resistance resources that enable individuals to cope with stress and maintain health. The implication here is that physical exercise may act as a health promoting factor, enhancing the ability of older adults to manage their health and reducing their reliance on medical services. Furthermore, the Health Belief Model (18) suggests that individuals who perceive themselves to be at lower risk are less likely to seek medical care. This is supported by the research, which identified perceived health status as a significant predictor of healthcare utilization (19). Additionally, the Self-Regulation Model of Health Behavior (20) posits that individuals’ interpretations of their symptoms and health status guide their health actions, which may explain why older adults who exercise regularly may experience fewer symptoms and have a more positive perception of their health, leading to a reduced need for medical services.

### 4.2 The mediating role of self-perceived physical health

The mediation analysis in this study revealed that self-perceived physical health significantly mediated the relationship between physical exercise and health care seeking behavior, with an indirect effect of −0.026, accounting for 29.89% of the total effect. This finding aligns with Dostálová et al.’s (21) observation of exercise-induced improvements in self-perceived health (*β* = 0.33, *p* = 0.07). It suggests that the perception of one’s physical health is a crucial factor influencing the decision to seek medical care. This aligns with the Self-Regulation Model of Health Behavior, which posits that individuals’ interpretations of their symptoms and health status guide their health actions (19). Our findings suggest that older adults who exercise regularly may experience fewer symptoms and have a more positive perception of their health, leading to a reduced need for medical services. Moreover, the Common Sense Model suggests that individuals use their own schema to interpret health threats, which may influence their health behavior (20). This is supported by the work of Shaelyn et al. (22), who found that older adults with a more positive self-assessment of their physical health were less likely to engage in avoidant health behaviors. Additionally, the Social Cognitive Theory emphasizes the role of self-efficacy in health behavior, suggesting that individuals who believe they can manage their health effectively are less likely to seek medical care (23). This is further supported by the study by Elavsky et al. (24), which reported that older adults with higher self-efficacy in physical activity had lower healthcare utilization. Lastly, the Transtheoretical Model suggests that individuals progress through stages of change, and those in later stages are more likely to engage in health-promoting behaviors, which may reduce their need for medical services (25). Furthermore, it is worth considering that self-perceived physical health may also influence preventive healthcare behaviors. Older adults with a positive health perception may be more inclined to engage in regular health check-ups or adopt healthier lifestyles, thereby reducing the occurrence of severe health issues and the subsequent need for extensive medical interventions.

### 4.3 The mediating role of self-perceived mental health

Our analysis revealed a statistically significant mediating role of self-perceived mental health in the relationship between physical exercise and health care seeking behavior (indirect effect = −0.008), accounting for 9.2% of the total effect. It suggests that the psychological benefits of exercise may contribute to a reduced need for medical services. This is supported by the literature on mental health and physical health comorbidity, which suggests that mental health issues can exacerbate physical health problems and increase the use of healthcare services (26). Our findings suggest that by improving mental health, physical exercise may reduce the exacerbation of physical health issues and decrease the need for medical interventions. Furthermore, the Biopsychosocial Model (27) emphasizes the impact of psychological factors on health outcomes. This is supported by the study by Adepoju (28), which noted that better mental health was associated with lower healthcare utilization among older adults. Additionally, the Social Support Theory suggests that social support can buffer the effects of stress and improve mental health, which may influence healthcare seeking behavior (29). This is further supported by the work of Hamid (30), who found that older adults with stronger social networks had better mental health and lower healthcare utilization. Lastly, the Stress-Buffering Model posits that social support can reduce the negative impact of stress on health, which may explain why older adults who exercise and have better mental health seek medical care less frequently (31). Moreover, it is worth considering that improved self-perceived mental health through regular physical exercise may also enhance older adults’ resilience and coping mechanisms. This, in turn, could reduce their perceived need for medical care, as they may feel more capable of managing minor health issues independently.

### 4.4 Age-related heterogeneity in the effects of physical exercise

The heterogeneity analysis in our study reveals significant differences in the impact of physical exercise on health care seeking behavior across different age groups of older adults. This finding is supported by the Selective Optimization with Compensation theory, which suggests that older adults adapt their behavior based on their age-related resources and needs (32). Our results contribute to the work of Jakovljevic et al. (33), who found age-related differences in the effects of physical activity on health outcomes. The practical implication is that public health interventions should consider the specific needs and capacities of different age groups when promoting physical activity. Furthermore, the Disengagement Theory suggests that older adults may withdraw from social roles and activities, including health-related behaviors, as they age (34). This is supported by the study by Shin (35), which reported that older adults in their 80s and above were less likely to engage in physical activity compared to younger older adults. Additionally, the Activity Theory posits that older adults maintain their mental health by remaining active and engaged in social roles (36), which may influence their health behaviors. This is further supported by the work of Yilmaz (37), who found that older adults who were more active had better health outcomes. Lastly, the Continuity Theory suggests that older adults tend to maintain their health behaviors from middle age into later life, which may explain the differences in the impact of physical exercise on health care seeking behavior across age groups (38). Furthermore, another possible explanation is that as age increases, other factors such as the presence of family members and economic status have a greater impact on older adults’ health care seeking behavior than their own health status. For example, some older adults may not be able to seek medical treatment voluntarily due to disability, lack of companionship, or poor economic conditions. Moreover, it is worth considering that older adults in more advanced age groups may benefit from alternative forms of low-impact physical activities, such as tai chi or water aerobics, which are better suited to their physical limitations (39).

This study makes significant contributions both theoretically and practically. First, it explores the impact of physical exercise on health care seeking behavior among older adults and reveals the mediating roles of physical and mental health, providing new insights into understanding health behaviors in older adults. The use of regression analysis and mediation tests provides preliminary evidence of the relationship between physical exercise and health care seeking behavior, offering a scientific basis for health promotion interventions. Second, the study highlights significant age-related differences in how physical exercise affects health care seeking behavior, enriching the existing literature on age-related health behaviors in older adults. To ensure the robustness of the findings, this study validated the results by substituting variables (such as sports venue and High-intensity exercise) in regression models and employed a dual machine learning model to test the stability of the results. The dual model, which combines traditional econometric methods with machine learning techniques, further confirmed the reliability and robustness of the conclusions.

However, there are certain limitations to this study. First, due to the cross-sectional design of the data, causality cannot be inferred, and future longitudinal studies should be conducted to establish causal relationships. Second, this study could not fully account for all potential confounding variables, such as participants’ medical history and social support networks, which may impact the accuracy of the results. Additionally, although heterogeneity analysis was conducted across different age groups, some groups, particularly those aged 80 and above, had small sample sizes, which may limit the generalizability of the findings for this subgroup. Lastly, although physical and mental health was analyzed as a mediating variable in this study, there are other variables such as diet, chronic diseases, daily medication, and medical history that can affect the physical exercise and health care seeking behavior of older adults. Future studies should further explore the mediating and moderating effects of these factors.

This study has several innovative aspects and advantages. First, compared with existing studies, our research not only confirms the impact of physical exercise on older adults’ health care seeking behavior but also further explores the mediating roles of physical and mental health, revealing the underlying mechanisms. This provides a deeper theoretical foundation for older adults’ health management. Second, in the robustness check, the high-intensity exercise variable has been introduced, verifying our findings from the perspective of different exercise types. This extends the analytical framework of existing studies. Moreover, this study utilizes nationally representative CGSS 2021 data, covering a broader older adults and conducting age-stratified analysis, with particular attention to the unique characteristics of individuals aged 80 and above, enhancing the practical significance of this findings. Additionally, two machine learning models, LASSO Regression and Ridge Regression, are also employed for supplementary analysis. These extended analyses not only strengthen the reliability of research conclusions but also provide more targeted references for public health policy formulation.

The health promotion intervention strategies of this study are as follows. Firstly, promoting regular physical activity programs should be prioritized. Community-based exercise initiatives, such as walking groups, tai chi, or water aerobics, should be tailored to older adults, particularly those aged 60–79, who demonstrated the strongest reduction in health care seeking behavior due to exercise. Incentivizing participation through subsidies or rewards (e.g., discounted gym memberships, health insurance benefits) could further encourage engagement. Additionally, improving access to safe and age-appropriate exercise venues—such as parks and senior fitness zones—is essential, especially in rural areas where environmental barriers may limit opportunities for physical activity. Secondly, integrating health education and self-efficacy building into intervention strategies is crucial. Workshops should be organized to help older adults understand the connection between exercise, self-perceived health, and reduced medical reliance, emphasizing the preventive benefits of physical activity against chronic diseases. Peer-led support groups, where older adults who have experienced positive health outcomes from exercise mentor their peers, can foster motivation and reinforce social support, aligning with Social Cognitive Theory principles. Thirdly, age-specific and personalized interventions should be implemented. For adults aged 60–69, moderate-intensity activities like brisk walking or cycling can help maintain physical health and delay age-related decline. For those aged 70–79, balance and flexibility exercises (e.g., yoga, resistance training) should be emphasized to reduce frailty and enhance mental well-being, given this group’s significant response to exercise. Adults aged 80 and above may benefit from low-intensity, supervised activities (e.g., chair exercises) combined with regular health monitoring to accommodate mobility limitations and comorbidities. Finally, addressing mental health mediation through mind–body programs and counseling services can amplify the benefits of physical activity. Incorporating mindfulness-based exercises (e.g., meditation, qigong) into fitness regimens can improve mental well-being and reduce anxiety-driven medical visits. Additionally, offering free or low-cost mental health screenings alongside exercise programs can help identify and address psychological barriers to self-care, ensuring a holistic approach to health management. By implementing these targeted strategies, policymakers and healthcare providers can effectively reduce unnecessary medical utilization among older adults while enhancing their overall well-being.

## 5 Conclusion

This study provides preliminary evidence that physical exercise is negatively associated with health care seeking behavior among older adults, and the higher the frequency of physical exercise, the greater the promotion effect on reducing health care seeking behavior. The findings demonstrate that self-perceived physical and mental health were identified as significant mediators in the relationship between physical exercise and health care seeking behavior. The subgroup analysis revealed age-related differences in the impact of physical exercise on health care seeking behavior. Older adults aged 70–79 years showed a stronger positive effect of physical exercise on health care seeking behavior compared to those in the 60–69 age group, while the effect for those aged 80 and above was not statistically significant. This suggests that the benefits of physical exercise on health behavior may vary across different age groups.

## Data Availability

The original contributions presented in the study are included in the article/supplementary material, further inquiries can be directed to the corresponding authors.
